# Histone variant H2A.Z regulates nucleosome unwrapping and CTCF binding in mouse ES cells

**DOI:** 10.1093/nar/gkaa360

**Published:** 2020-05-11

**Authors:** Zengqi Wen, Liwei Zhang, Haihe Ruan, Guohong Li

**Affiliations:** 1 National Laboratory of Biomacromolecules, CAS Center for Excellence in Biomacromolecules, Institute of Biophysics, Chinese Academy of Sciences, Beijing 100101, China; 2 University of Chinese Academy of Sciences, Beijing, China

## Abstract

Nucleosome is the basic structural unit of chromatin, and its dynamics plays critical roles in the regulation of genome functions. However, how the nucleosome structure is regulated by histone variants *in vivo* is still largely uncharacterized. Here, by employing Micrococcal nuclease (MNase) digestion of crosslinked chromatin followed by chromatin immunoprecipitation (ChIP) and paired-end sequencing (MNase-X-ChIP-seq), we mapped unwrapping states of nucleosomes containing histone variant H2A.Z in mouse embryonic stem (ES) cells. We found that H2A.Z nucleosomes are more enriched with unwrapping states compared with canonical nucleosomes. Interestingly, +1 H2A.Z nucleosomes with 30–80 bp DNA is correlated with less active genes compared with +1 H2A.Z nucleosomes with 120–140 bp DNA. We confirmed the unwrapping of H2A.Z nucleosomes under native condition by re-ChIP of H2A.Z and H2A after CTCF CUT&RUN in mouse ES cells. Importantly, we found that depletion of H2A.Z results in decreased unwrapping of H3.3 nucleosomes and increased CTCF binding. Taken together, through MNase-X-ChIP-seq, we showed that histone variant H2A.Z regulates nucleosome unwrapping in vivo and that its function in regulating transcription or CTCF binding is correlated with unwrapping states of H2A.Z nucleosomes.

## INTRODUCTION

The genome of eukaryotic cells is packaged with histones to form chromatin in the nucleus. Chromatin is the template for all the DNA metabolism processes, such as transcription, DNA replication and repair. Nucleosome is the basic unit of chromatin and plays critical roles in the regulation of genome functions. An intact nucleosome is composed of an octamer of histones, which contains two copies of each of H2A, H2B, H3 and H4, and 146 base pairs (bp) of DNA. The crystal structure of the nucleosome core particle showed that the DNA was wrapped on the octamer by about 1.65 superhelix turn in a left-hand manner with periodic interaction with histones (1). During the nucleosome assembly mediated by salt dialysis *in vitro*, an (H3-H4)_2_ tetramer bind DNA first to form a tetrasome wrapping about 80 bp DNA, then two H2A–H2B heterodimers were added sequentially to form an intact nucleosome (2). However, nucleosomes are not static entities, but can undergo spontaneous structural transitions, such as DNA breathing (transient release of DNA ends) and open state (transient opening of the interface between histone sub-complexes) (2), which could lead to the formation of unwrapped nucleosomes. Recent studies have revealed additional insights into the variations and regulation of nucleosome structure *in vitro*. A dynamic intermediate nucleosome structure called prenucleosome, which consists of a histone octamer wrapped by ∼80 bp of DNA, was reported and it can be converted into intact nucleosomes by histone chaperone (3). During the nucleosome assembly and remodeling, two neighboring nucleosomes can collapse with each other and result in loss of a H2A–H2B dimer and formation of the hexasome (4,5). The nucleosomal DNA can unwrap asymmetrically and directionally under tension, which is regulated by the flexibility of DNA sequence and histone chaperone FACT (6,7). Whereas variations and regulation of nucleosome unwrapping have been demonstrated *in vitro*, the landscape and regulation of unwrapped nucleosomes *in vivo* are much less characterized.

The unwrapping states of nucleosomes may exit due to nucleosome dynamics and maturation during transcription and replication *in vivo*. RNA polymerase II is a strong remodeler of nucleosomes *in vivo*, and passage of RNA polymerase II can open nucleosomes and generate hexasome or other unwrapping states of nucleosomes (8–10). During DNA replication, nucleosomes are disrupted ahead of the replication fork and are reassembled behind the replication fork (11,12). Apart from assembly and disassembly dynamics, nucleosome structure is regulated by other factors *in vivo*, such as histone variants, chaperones and chromatin remodelers (2). Therefore, the nucleosome states in *vivo*, especially the states of unwrapped nucleosomes, could even be more diversified than in *vitro*. Chromatin immunoprecipitation-exonuclease digestion (ChIP-exo) has been used to analyze the organization of individual histones within a nucleosome at genome-wide in yeast. Subnucleosome structures and asymmetries of histone compositions were found to be widespread across the yeast genome (13). Micrococcal nuclease (MNase) has long been used to analyze nucleosome positioning at single genomic loci and at genome-wide (14). Recently, combining with paired-end sequencing of total protected DNA after digestion, MNase-seq has been used to analyze the genome-wide chromatin organization, including both nucleosomal and subnucleosomal particles, in budding yeast (15,16), mouse ES cells and sperm (17), and *Drosophila* cells (18). However, as the protection (especially subnucleosomal protection) from MNase digestion can also be attributed from other chromatin binding factors (15,16), there is a limitation of this method to analyze the *in vivo* nucleosomal states directly, particular the unwrapped nucleosomes. Here, we performed MNase digestion of crosslink chromatin followed with ChIP and paired-end sequencing (MNase-X-ChIP-seq) to analyze the genome-wide unwrapping states of H2A.Z nucleosomes in mouse ES cells. Our results showed that H2A.Z is enriched with nucleosome unwrapping compared with canonical nucleosomes, and H2A.Z could function in gene regulation and CTCF binding regulation through modulating the unwrapping states of nucleosomes.

## MATERIALS AND METHODS

### Cell culture and siRNA transfection

Mouse ES cells were cultured in the medium with 80% DMEM (EmbryoMax, SLM-220-B), 15% FBS (Hyclone, SH30070.03), Nonessential amino acids (EmbryoMax, TMS-001-C), 2-Mercaptoethanol (EmbryoMax, ES-007-E), l-glutamine (EmbryoMax, TMS-002-C), Nucleosides (EmbryoMax, ES-008-D), Pen/Strep (EmbryoMax, TMS-AB-2C) and 1000 U/ml leukemia inhibitory factor (LIF) (ESGRO, ESG1107) in standard incubator with 5% CO_2_ at 37°C. Plasmids or siRNA oligos were transfected into mouse ES cells by Lipofectamine 3000 (Invitrogen) according to the manufacturer's instructions.

### H2A.Z knock down in mES cells

To generate H2A.Z depletion cells, H2A.Z was knocked down by the siH2A.Z oligo: 5′-GGTAAGGCTGGAAAGGACT-3′. Knock down efficiency was confirmed by western blot.

### MNase digestion facilitated ChIP coupled with pair-end sequencing (MNase-X-ChIP-seq)

For MNase X-ChIP, mouse ES cells were crosslinked with 1% formaldehyde in DMEM for 10 min at room temperature, then quenched by 125 mM glycine. Cells were washed with cold DPBS for twice, and then resuspended in lysis buffer (10 mM Tris [pH 7.5], 10 mM NaCl, 2 mM MgCl_2_, 0.5% NP-40, 1 mM CaCl_2_) (19) with protease inhibitors (Roche) and incubated for 15 min at 4°C. Then the cells were pre-warmed at 37°C for 3 min, and digested with 0.5 U/ml MNase (Sigma, N3755). 10 mM EDTA was added to stop the digestion. Then *N*-lauroyl-sarcosine was added at final concentration of 0.5%. The cell pellets were sonicated using a Bioruptor UCD-200 (Diagenode), and the chromatin particles before crosslink reversion are ∼1000–2000 bp after resolved on 1% agarose, which is the typical chromatin particle size for ChIP-seq.

For H2A.Z, H2A or H3 ChIP, chromatin was first incubated with H2A.Z (Abcam, ab4174), H2A (Abcam, ab18255) or H3 (Abcam, ab1791) antibody in RIPE-150 (50 mM Tris–HCl, 150 mM NaCl, 1 mM EDTA, 0.5% Triton X-100, protease inhibitors) at 4°C, then the BSA blocked protein A/G Dynabeads were added and incubated overnight at 4°C. The Dynabeads were washed by RIPE-150 with 0.1% SDS for 5 times, and eluted with Direct Elution Buffer (10 mM Tris–HCl pH 8, 0.3 M NaCl, 5 mM EDTA pH8, 0.5% SDS).

The ChIPed DNAs were extracted using a standard phenol-chloroform extraction procedure. For paired-end sequencing, libraries without size selection were prepared as described in (15) using NEBNext Ultra DNA Library Prep Kit for Illumina (E7370L) and were sequenced using Illumina HiSeq X-10 or NovaSeq 6000 platform.

### CTCF ChIP-seq

Chromatin was crosslinked with 1% formaldehyde. ChIP was performed as previously described (20) with the following modifications. Dynabeads and CTCF antibody (Cell Signaling, 2899) was incubated in RIPA-150 buffer (50 mM Tris–HCl, 150 mM NaCl, 1 mM EDTA, 1% Triton X-100, protease inhibitors). Then Dynabeads/antibody complexes were incubated with chromatin in RIPA-150 buffer, and washed with RIPA-150 buffer (with 0.1% SDS) for twice, RIPA-500 buffer (50 mM Tris–HCl, 500 mM NaCl, 1 mM EDTA, 1% Triton X-100, 0.1% SDS, protease inhibitors) for twice, RIPA-LiCl (50 mM Tris–HCl pH 8, 1 mM EDTA pH 8, 1% Nonidet *P*-40, 0.7% sodium deoxycholate, 0.5M LiCl_2_) for once and 1× TE Buffer, pH 8.0 for twice. Libraries were prepared and sequenced using Illumina HiSeq2000 platform, and sequencing data were processed as previously described (20).

### CTCF CUT&RUN and re-ChIP with H2A or H2A.Z

CTCF CUT&RUN was performed as described in (21). Briefly, cells were incubated in NE1 (20 mM HEPES–KOH, PH 7.9; 10 mM KCl, 1 mM MgCl_2_, 0.1% Triton X-100, 20% Glyceral) on ice for 10 min. The nuclei were spin down at 600 × g for 3 min at 4°C, and re-suspended in Buffer I (20 mM HEPES, PH 7.5; 150 mM NaCl, 2 mM EDTA, 0.5 mM spermidine, 0.1% BSA) on ice for 5 min. The nuclei were spin down at 600 × g for 3 min at 4°C, and washed once by Buffer II (20 mM HEPES, PH 7.5; 150 mM NaCl, 0.5 mM spemidine, 0.1% BSA) and re-suspended in Buffer II. 15 ul CTCF antibody (Millipore, 07-449) was used for each 2 × 10^7^ cells and incubated at 4°C for 2 h on a mixing platform. The nuclei were washed with Buffer II for three times and re-suspended at 1 × 10^7^ cells/600 ul Buffer II /6 ug pA-MNase (a kind gift from Dr. Bing Zhu, Institute of Biophysics, CAS), and incubated at 4°C for 1 h on a mixing platform. The nuclei were washed with Buffer II for three times and re-suspended at 1 × 10^7^ cells/1 ml Buffer II with 2 mM CaCl_2_. The digestion was performed at 25°C for 20 min without mixing. The digestion was stopped with 10 mM EGTA. The nuclei were incubated on a thermal incubator at 37°C for 30 min with 900 rmp angitation. The nuclei were centrifuged at 15 000 rpm for 10 min at 4°C. The supernatant were taken out without disturbing the pellet. 10% percentage of the supernatant was kept for input of CTCF CUT&RUN. Protein A and Protein G Dynabeads were blocked with 0.1% BSA and then incubated with H2A.Z antibody (Abcam, ab4174) or H2A antibody (Abcam, ab18255). The resulting Dynabeads/antibody complexes were incubated with the remaining supernatant at 4°C for 6 h on a mixing platform. The beads were washed for three times by Buffer II with 0.1% Triton X-100. The chromatin was eluted with Direct Elution Buffer (10 mM Tris–HCl pH 8, 0.3 M NaCl, 5 mM EDTA pH 8, 0.5% SDS). The ChIPed DNAs were extracted using a standard phenol-chloroform extraction procedure. For paired-end sequencing, libraries without size selection were prepared as described in (15) using NEBNext Ultra DNA Library Prep Kit for Illumina (E7370L) and were sequenced using Illumina HiSeq X-10 or NovaSeq 6000 platform.

### Sequencing data analysis

Paired-end reads were trimmed for adaptor sequence using cutadapt (22) with parameters: -a AGATCGGAAGAGCACACGTCTGAACTCCAGTCAC -A AGATCGGAAGAGCGTCGTGTAG- GGAAAGAGTGTAGATCTCGGTGGTCGCCGTATCATT -e 0.1 -n 2 -m 35 -q 30 –pair-filter = any, and then mapped to mm9 using Bowtie2 (23) with parameters: -3 3 –no-discordant –no-mixed. The concordantly mapped read pairs were filtered using a python script to retain those with mapq > 10. H2A.Z peaks are first called using MACS2 (24) with parameters as: -t -c -n –format BEDPE -m 5 50 -q 0.05 -g mm –broad, then peaks with mfold > = 3 and *q* ≤ 0.001 are selected. Enriched peaks were detected using MACS2 with default parameters. The overlapping between peaks was analyzed with the BEDTools software (25). The reads within 1 kb regions of peaks or within 200 bp regions of +1 nucleosomes were counted using a Python script, and the read ratio of 35–80 bp, 81–100, 101–120, 121–140 and 141–168 bp DNA were calculate. A Python script (written referring to the Perl script provided by Drs Jorja G. Henikoff and Steven Henikoff) was used to perform V-plot analysis and the signals were normalized to centers of fragments per billion read pairs (CPB) at each base pair, or aggregate according to fragment groups. Hierarchical clustering were performed by function ‘hclust()’ from R package ‘stats’ after the ratios were centered by median and scaled by Median absolute deviation within each fragment group. Heatmap were generated using function ‘heatmap.2()’ from R package ‘gplots v3.0.1’ (https://CRAN.R-project.org/package=gplots). Motif analysis and gene ontology analysis were performed with HOMER (26). Other plots were generated by R (http://www.r-project.org/) or Microsoft Excel.

## RESULTS

### H2A.Z is enriched with nucleosome unwrapping

MNase-X-ChIP-seq has been used to achieve high resolution mapping of the binding sites of Pol II, Chd1 and CTCF in *Drosophila* cells (27,28). Here we used this method to map the unwrapping states of nucleosome in mouse ES cells. Chromatin was first crosslinked with formaldehyde, then digested with MNase and solubilized by minimal sonication (Supplementary Figure S1A). After Chromatin immunoprecipitation (ChIP), the ChIPed DNA was subjected to paired-end sequencing without size selection. The read pairs were mapped to the mouse reference genome (mm9) using Bowtie2, and only read pairs with mapping quality higher than 10 (mapq > 10) were retained for further analyses. We counted the frequency of the fragment length of ChIPed DNA per base pair from 0 to 200 bp to acquire the fragment length profiles (FLPs) of histones.

**Figure 1.**
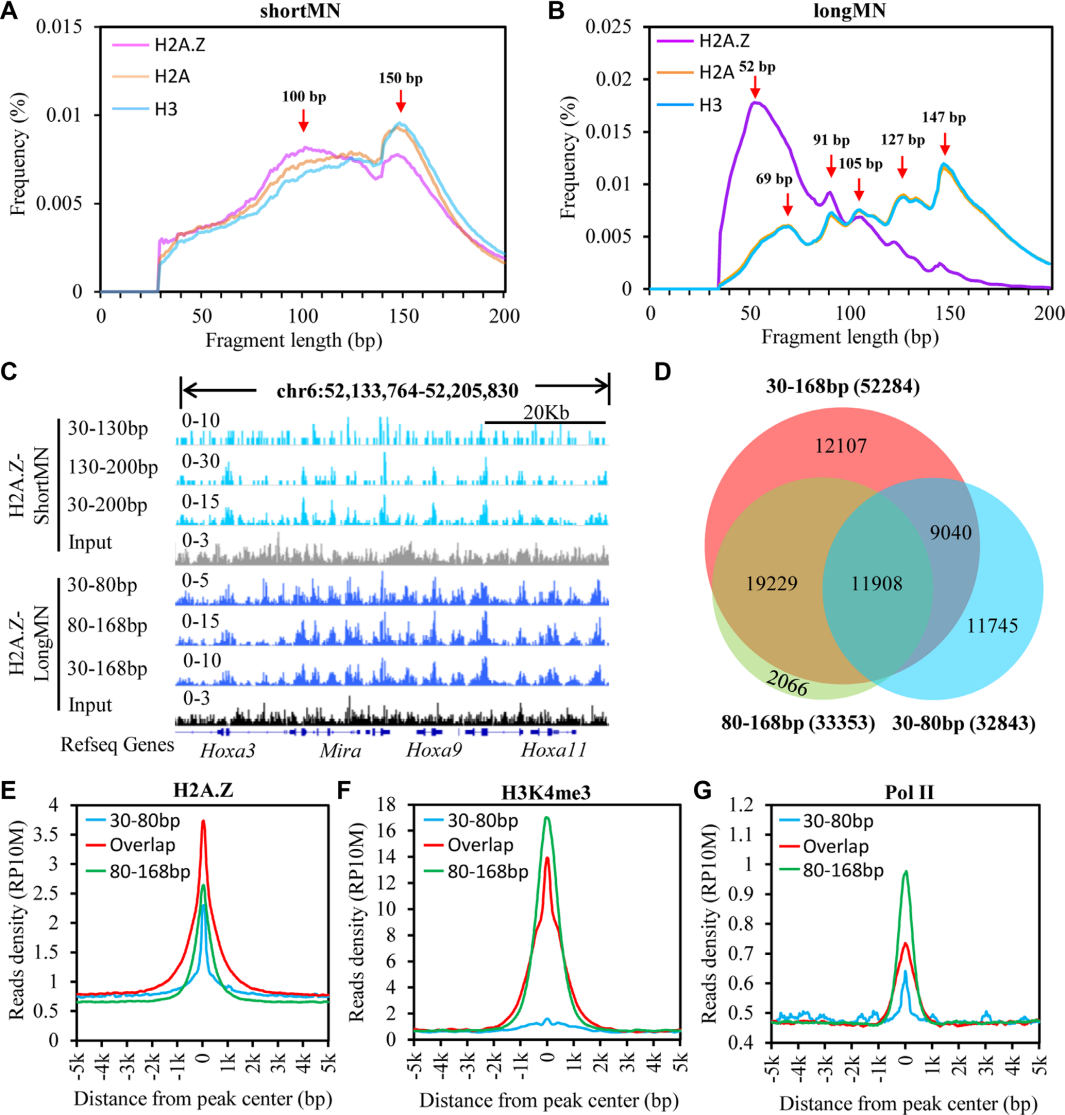
H2A.Z is enriched with nucleosome unwrapping. (**A**) Histogram shows the fragment length profiles (FLPs) of DNA fragments ChIPed by H2A.Z, H2A or H3 under ‘shortMN’ digestion condition. The red arrows indicate the major fragment length peaks. (**B**) Histogram shows the FLPs of DNA fragments ChIPed by H2A.Z, H2A or H3 under ‘longMN’ digestion condition. The red arrows indicate the major fragment length peaks. (**C**) Genome tracks show the distribution of H2A.Z ChIPed fragments under ‘shortMN’ and ‘longMN’ digestion conditions. The signals for all the tracks are normalized by reads per million (RPM) per 10 bp bin. (**D**) Venn plot shows the overlapping between peaks (*n* = 32843) of 30–80 bp DNA fragments and peaks (*n* = 33353) of 80–168 bp DNA fragments of H2A.Z nucleosomes. (E–G) Histograms show the distribution of H2A.Z (**E**), H3K4me3 (**F**) and Pol II (**G**) around 30–80bp specific peaks (*n* = 20935), Overlap peaks (*n* = 11 908) and 80–168 bp specific peaks (*n* = 21 445).

We performed MNase-X-ChIP-seq for histone variant H2A.Z and canonical histones H2A and H3 under both ‘shortMN’ (4 min digestion) and ‘longMN’ (32 min digestion) conditions (Supplementary Figure S1A). We found that under both conditions, the sonication treatments did not change the FLP much (Supplementary Figure S1B and C). Under ‘shortMN’ condition, we found that canonical nucleosomes showed a sharp peak at ∼150 bp and a broad peak at 80–140 bp (Figure 1A), suggesting that canonical nucleosomes in vivo may have two different populations of nucleosome structure. Interestingly, we found that H2A.Z nucleosomes showed a lower peak at 150 bp and higher peak at ∼100 bp compared with canonical nucleosomes (Figure 1A). This result suggested that H2A.Z is more enriched with unwrapped nucleosomes than the canonical histones. Compared with the FLPs under ‘shortMN’ condition, the FLPs of both H2A.Z nucleosomes and canonical nucleosomes changed dramatically under ‘longMN’ condition (Figure 1B). The FLPs of H2A and H3 are almost identical, with the summit at 147 bp, which corresponds to an intact nucleosome core particle. Moreover, we also observed clear sub-peaks at 127, 105, 91 and 69 bp, which may represent different unwrapping states of canonical nucleosomes *in vivo*. Similarly, distinct states of MNase protection were also observed in the FLP of the ‘longMN’ input (Supplementary Figure S1C). Previously, in Drosophila S2 cells, the 125, 103 and 90 bp DNA fragments from MNase digestion of chromatin have been proposed to be derived from subnucleosomal protection from MNase digestion (18). Together, these results suggest that canonical nucleosomes have multiple unwrapping states in vivo. Moreover, our results showed that the small fragments (<80 bp) could also be derived from protection by the unwrapped nucleosomes, apart from chromatin binding factors, such as chromatin remodelers and transcription factors as reported previously (15,16). Few peaks can be called for any fragment groups when analyzed by MACS2 (24) (data not shown), and all the fragments of canonical histones do not show specific enrichment as displayed by the genome browser (Supplementary Figure S1D, E). Surprisingly, we found that H2A.Z nucleosomes have a dramatically different FLP from the canonical nucleosomes, with a major peak at 30–80 bp and far lower signal of larger DNA fragments (Figure 1B). This result confirms that H2A.Z nucleosomes are more unwrapped than canonical nucleosomes in mouse ES cells, as observed under ‘shortMN’ digestion. As we can resolve more unwrapping states of nucleosomes under ‘longMN’ digestion than under ‘shortMN’ digestion, we will focus on ‘longMN’ digestion in the following analyses.

We separated the H2A.Z ChIPed DNA under ‘longMN’ condition into 30–80 and 80–168 bp groups. We found the genome-wide enrichment patterns of these two group DNA fragments are generally similar to that of total H2A.Z (Figure 1C). Next, we defined 32,843 , 33 353 and 52 284 enriched regions for the 30–80, 80–168 bp and total H2A.Z ChIPed DNA, respectively, by MACS2 (24). We found that most of the 30–80 bp peaks and 80–168 bp peaks overlapped with total peaks (Figure 1D). These results suggest that the small DNA fragments represent unwrapped H2A.Z nucleosomes. We further analyzed the chromatin modifications of the 30–80 bp specific peaks (*n* = 20 935), overlap peaks (*n* = 11 908) and 80–168 bp specific peaks (*n* = 21 445). We found that the overlap peaks showed higher level of H2A.Z (Figure 1E), and the overlap peaks and 80–168bp specific peaks have higher level of H3K4me3 and Pol II than 30–80bp specific peaks (Figure 1F, G), suggesting that H2A.Z nucleosomes at the promoters are less unwrapped than other H2A.Z nucleosomes.

We further analyzed the FLPs of canonical histones at the genomic regions decorated by different chromatin modifications, including H3K4me1, H3K4me3, H3K27ac, H3K36me3 and H3K27me3. We found that all the analyzed genomic regions show similar FLPs as the bulk canonical histones (Supplementary Figure S1F). However, to more directly evaluate the effects of histone modifications on the nucleosome unwrapping in vivo, MNase-X-ChIP-seq of specific histone modification is required to analyze the FLP of the modified nucleosomes.

### Unwrapping of H2A.Z nucleosomes at the promoters

**Figure 2.**
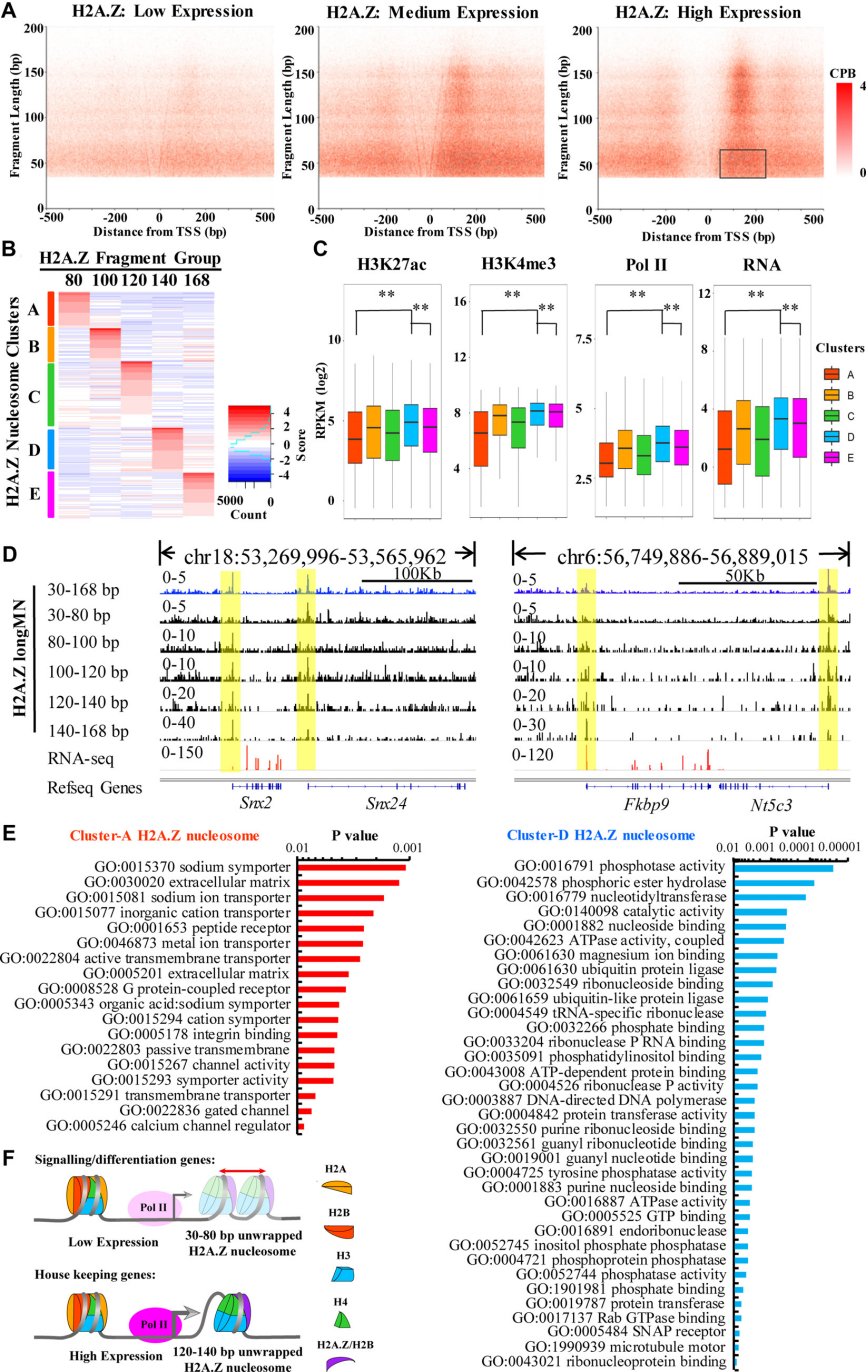
Unwrapping of H2A.Z nucleosomes at the promoters. (**A**) V-plots show the FLPs of H2A.Z ChIPed DNA fragments around the transcription start sites (TSSs) of low, medium and high expressed genes. The grey box highlights the signals indicating diffusing unwrapped +1 H2A.Z nucleosomes. Refer to Figure 1G for the interpretation of V-plot. (**B**) Hierarchical clustering of +1 H2A.Z nucleosomes based on the ratios of the five DNA fragment groups within each +1 H2A.Z nucleosome. The ratios were centered by median and scaled by median absolute deviation within each fragment group for clustering. Group 80, 100, 120, 140, 168 include fragments of 30–80, 80–100, 100–120, 120–140 and 140–168 bp, respectively. (**C**) Boxplots show the levels of H3K27ac, H3K4me3, Pol II and gene expression of promoters co-localized with each cluster of H2A.Z nucleosome. ** indicates *P*-value < 0.01. (**D**) Genome tracks of the H2A.Z ChIPed DNA fragments show the depletion of 30–80 bp DNA fragments of H2A.Z nucleosomes at the high expressed genes *Snx2* and *Fkbp9*, and the presence of 30–80 bp DNA fragments of H2A.Z nucleosomes at the low expressed genes *Snx24* and *Nt5c3*. The signals for all the tracks are normalized by reads per million (RPM) per 10 bp bin. (**E**) Gene ontology analysis revealed the enriched functions of the genes co-localized with Cluster-A or Cluster-D unwrapped H2A.Z nucleosomes. (**F**) A diagram shows the working model that the function of the +1 H2A.Z nucleosomes in transcription regulation is correlated with its unwrapping states. The +1 H2A.Z nucleosomes with the highest ratio of 30–80 bp DNA fragments are associated with less active genes, such as the genes involved in signaling transduction. The +1 H2A.Z nucleosomes with highest ratio of 120–140 bp DNA fragments are associated with genes involved in housekeeping functions.

To study the unwrapping states of H2A.Z nucleosomes at the promoters, we analyze H2A.Z ChIPed DNA by V-plot as previously described (refer to Supplementary Figure S1G for detailed interpretation of V-plot used in this study) (15). We further separated the 80–168 bp DNA of H2A.Z nucleosomes into 80–100, 100–120, 120–140 and 140–168 bp groups according to the sub-peaks in the FLPs of canonical nucleosomes (Figure 1B). We found that, irrespective of the unwrapping states, H2A.Z nucleosomes are well phased and enriched at +1 nucleosome, especially at high expression genes (Figure 2A and Supplementary Figure S2A), whereas canonical nucleosomes are less enriched at the promoter regions (Supplementary Figure S2B, C). Moreover, the signal of 30–80 bp DNA fragments ChIPed by H2A.Z is higher than that of other fragment groups (Supplementary Figure S2A), suggesting that unwrapped H2A.Z nucleosomes with 30–80 bp DNA is prevalent at the promoters. At the nucleosome depleted region (NDR) of promoters, we observed unwrapped H2A.Z nucleosomes (30–80 bp DNA), especially at the NDR of medium expression genes (Figure 2A and Supplementary Figure S2A). In the input of MNase-X-ChIP, we also found enriched signal of small DNA fragments at the NDR (Supplementary Figure S2D). These results are consistent with the observation of fragile nucleosomes at the NDR in yeast (29,30), suggesting that the fragility of these nucleosomes is due to nucleosome unwrapping.

To further analyze the correlation between the unwrapping states of +1 H2A.Z nucleosomes and transcription, we first identified the H2A.Z nucleosome centers using all of the H2A.Z ChIPed DNA fragments by iNPS (31), then we selected the H2A.Z nucleosome centers within 50–200 bp downstream of the TSSs as +1 H2A.Z nucleosomes. We further calculated the ratio of 30–80, 80–100, 100–120, 120–140 and 140–168 bp groups of DNA fragments ChIPed by H2A.Z within the 200 bp region of each nucleosome center. We found that the ratio of the 30–80 bp fragments is negatively correlated with gene expression (Spearman correlation, rho = −0.26, *P* = 7.5e−78) (Supplementary Figure S2E.1), whereas the ratios of the 120–140 bp fragments and 140–168 bp fragments are positively correlated with gene expression (Spearman correlation, rho = 0.22, *P* = 3.6e−42 for 120–140 bp DNA; Spearman correlation, rho = 0.30, *P* = 8.3e−74 for 140–168 bp DNA) (Supplementary Figure S2E.4, S2E.5). These results suggest that the function of +1 H2A.Z nucleosomes in transcription is correlated with their unwrapping states. We further normalized the ratio within each group and performed hierarchical clustering using the normalized scores. The +1 H2A.Z nucleosomes were clustered into five classes as a result (Figure 2B). Cluster-A, Cluster-B, Cluster-C, Cluster-D and Cluster-E +1 H2A.Z nucleosomes are characterized by relatively higher enrichment of 30–80, 80–100, 100–120, 120–140 and 140–168 bp DNA fragments than other clusters, respectively (Supplementary Figure S2F). However, in all the five defined clusters, the ratio of the 30–80 bp fragments is still higher than other groups (Supplementary Figure S2F). Thus, we interpret the Clusters B-E as dynamic unwrapping H2A.Z nucleosomes that have relative higher probability of transition from the unwrapping states with 30–80 bp DNA to unwrapping states with 80–100, 100–120, 120–140 and 140–168 bp DNA, respectively.

Next, we compared the epigenetic features of the five unwrapping states of H2A.Z nucleosomes. We found that Cluster-A and Cluster-D unwrapped H2A.Z nucleosomes are correlated with the lowest and the highest levels of H3K4me3, H3K27ac, Pol II and gene expression, respectively (Figure 2C). As is shown in Figure 2D, the presence of unwrapped H2A.Z nucleosomes with 30–80 bp DNA is associated with low gene expression (*Snx24* and *Nt5c3*); and the absence of unwrapped H2A.Z nucleosomes with 30–80 bp DNA is associated with high gene expression (*Snx2* and *Fkbp9*). These results confirm that the regulatory function of the +1 H2A.Z nucleosomes in transcription is correlated the unwrapping states. To test whether the Cluster-A and the Cluster-D unwrapped H2A.Z nucleosomes are involved in the regulation of genes with distinct biological functions, we performed Gene Ontology analysis with the genes associated with Cluster-A and Cluster-D H2A.Z nucleosomes by HOMER (26). Intriguingly, we found that the Cluster-A H2A.Z nucleosomes are enriched with the genes associated with ion transporter/channel activity and genes essential for signaling transduction, such as ‘G-protein coupled receptor signaling’ (Figure 2E, refer to Supplemental Table S1 for a full list of GO terms), whereas the Cluster-D H2A.Z nucleosomes are enriched with the genes essential for housekeeping functions, such as ‘phosphatase activity’, ‘nucleotidyltransferase’ and ‘nucleoside binding’ (Figure 2E, refer to Supplemental Table S2 for a full list of GO terms). We also analyzed the motif within the core promoter regions associated with group-A or group-D +1 H2A.Z nucleosomes by HOMER (26). We found that promoters associated group-A H2A.Z nucleosomes are enriched with motifs of cell type specific transcription factors, such as Ascl1, Hnf1, Pax7 and E2A (Supplemental Table S3). Ascl1 is reported to be involved in neuronal commitment and differentiation (32). This result is in agreement with the gone ontology analysis of promoters associated group-A H2A.Z nucleosomes. Promoters associated with group-D H2A.Z nucleosomes are enriched with GC-rich motifs (Supplemental Table S4), such as SP1, SP2, SP5, KLF3, KLF4, KLF5, whose functions are mostly non-cell type specific. This result is also consistent with that group-D H2A.Z nucleosomes are enriched of housekeeping genes, whose promoters are GC-rich (33,34). These results suggest that the function of +1 H2A.Z nucleosomes in regulating transcription and the cell identity of mESC is correlated with the unwrapping states (Figure 2F).

### Unwrapping of H2A.Z nucleosomes at the CTCF binding sites

**Figure 3.**
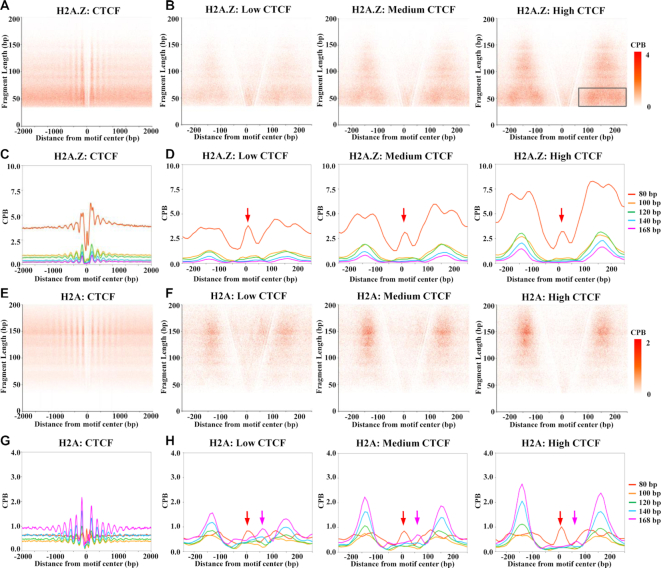
Unwrapping of H2A.Z nucleosomes at the CTCF binding sites. (**A, E**) V-plots show the FLPs of H2A.Z (A) and H2A (E) ChIPed DNA around 4 kb regions of CBS sites. The CTCF peaks are orientated and centered on the CTCF binding motif 5′-CCACNAGGTGGCAG-3′ in Figures 3–5, Supplementary Figure S3-S4. (B, F) V-plots show the fragment length distribution of H2A.Z (**B**) and H2A (**F**) ChIPed DNA around 500 bp regions of CBS sites with low, medium and high CTCF binding. Two populations of unwrapped H2A.Z nucleosomes with 30–80 bp DNA (indicated by the signals highlighted in the grey box) can be observed on each of the two nucleosomes immediately flanking CBSs. (C, G) Meta profiles show the reads density of the five fragment groups of H2A.Z (**C**) and H2A (**G**) ChIPed DNA around 4 kb regions of CBS sites. (D, H) Meta profiles show the reads density of H2A.Z (**D**) and H2A (**H**) ChIPed DNA around 500 bp regions of CBS sites with low, medium and high CTCF binding. Red arrows in Figure D and Figure H indicate moderate signals of unwrapped H2A.Z or H2A nucleosomes with 30–80 bp DNA at the center of CBSs. Cyan arrows in Figure H indicate moderate signals of intact H2A nucleosomes between CBSs the downstream nucleosome.

Previously, it has been shown that nucleosomes are well-phased around the CTCF binding sites (CBSs) (35), and are normally depleted at the center of CBS (17,36). To study the unwrapping states of H2A.Z nucleosomes at the CBSs, we mapped the CBSs by CTCF crosslink ChIP-seq. We defined the direction of CBSs according to the motif consensus (5′-CCACNAGGTGGCAG-3′) and centered the CBSs according to the CTCF motif (JASPAR MA0139.1) using HOMER (26). Then we analyzed the distribution of H2A.Z ChIPed DNA fragments by V-plot around the motif centered CBS. We found that, similar to FLP of total H2A.Z ChIPed DNA (Figure 1B), H2A.Z ChIPed DNA showed abundant signals of short fragments around the CBSs (Figure 3A, B). For both H2A.Z nucleosomes and canonical nucleosomes, they are well-phased around the CBSs (Figure 3A, C, E, G and Supplementary Figure S3A, C). After quantify the signal according to the five fragment groups, we found that the signals of all five fragment groups at the CBSs flanking nucleosomes are positively correlated with CTCF binding (Figure 3B, D, F, H and Supplementary Figure S3B, D). For H2A.Z nucleosomes, we found that the 30–80 bp group of DNA fragments has the highest signal compared with other fragment groups (Figure 3B, D). These results suggested that H2A.Z nucleosomes around CBSs are dominantly in the unwrapping state. In contrast, canonical histones showed higher signals of large DNA fragments (120–169 bp) than small DNA fragments (30–120 bp) (Figure 3E–H, and Supplementary Figure S3A–D). Interestingly, in contrast to the diffuse distribution at the +1 nucleosome, the 30–80 bp DNA fragments of H2A.Z nucleosomes show a strong bimodal pattern at each of the nucleosomes immediately flanking the CBSs (Figure 3B, D), which are positively correlated with CTCF binding activities. However, this pattern is not observed for canonical nucleosomes (Figure 3F, H and Supplementary Figure S3B, D). This bimodal pattern promotes us to hypothesis that H2A.Z–H2B dimers of the H2A.Z nucleosomes immediately flanking the CBSs may detach from the (H3–H4)_2_ tetramers to form specific unwrapped nucleosomes (Figure 5I).

We also observed moderate signals of 30–80 bp DNA fragments of both H2A.Z nucleosomes (Figure 3D, indicated by red arrows) and canonical nucleosomes (Supplementary Figure S3D, indicated by red arrows) at the center of CBSs. Of note, by a histone H4 based chemical cleavage approach, high nucleosome occupancies were also observed at the CTCF binding sites in a previous study (37). Thus, our results suggested that these H4 signals are likely contributed by the unwrapped nucleosomes, which cannot be detected by analyzing large fragments (longer than 140 bp) from MNase-seq (17). As shown by the input signal of MNase-X-ChIP, whereas 30–80 bp fragments are highly enriched at the center of CBS, the DNA fragments lager than 80 bp show little signal at the center of CBS (Supplementary Figure S3E–H). In addition, modest enrichments of the 140–168 bp DNA fragments of canonical nucleosomes were asymmetrically distributed downstream of the CBS, and this nucleosomal signal is anti-correlated with the strength of CTCF binding (Figure 3H and Supplementary Figure S3D, indicated by the magenta arrows). These signals of unwrapping nucleosomes at the center of CBSs and canonical nucleosome signals downstream of the CBS suggested that nucleosomes may compete with CTCF for the binding of CBSs (Figure 5I).

### H2A.Z regulates the unwrapping of H3.3 nucleosomes

**Figure 4.**
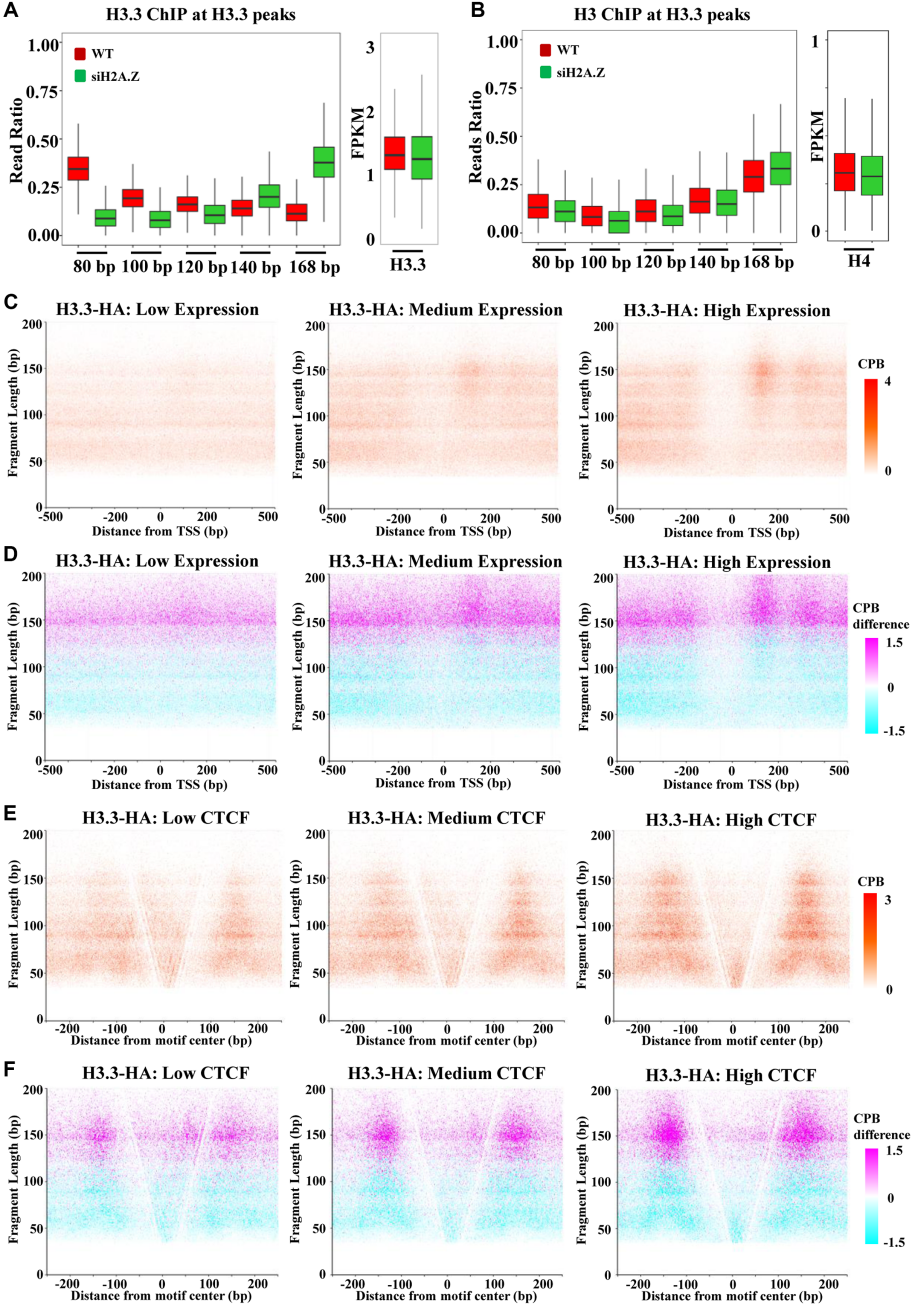
H2A.Z regulates the unwrapping of H3.3 nucleosomes. (A, B) Boxplots show the change of the unwrapping states of H3.3 nucleosomes (**A**) or H3 nucleosomes (**B**) at the H3.3 peak regions (*n* = 13 226). (C, E) V-plots show the fragment length distribution of H3.3–HA ChIPed DNA fragments around the transcription start sites (TSSs) of low, medium and high expression genes (**C**) or around CBS sites with low, medium and high CTCF binding (**E**). (D, F) Difference-V-plots show the changes of fragment length distribution of H3.3-HA ChIPed DNA fragments after H2A.Z knockdown cells around the transcription start sites (TSSs) of low, medium and high expression genes (**D**) or around CBS sites with low, medium and high CTCF binding (**F**). To plot a difference-V-plot, the fragment length distribution of both wild type and H2A.Z knockdown cells was counted as matrix (normalized to centers per billion fragments (CPB)) as the V-plot; and then each of the data points in H2A.Z knockdown matrix was subtracted by the corresponding data point (with same x-axis value and y-axis value) in wild type matrix to derive the CPB difference. Colors magenta and cyan indicate increased and decreased signals after H2A.Z knockdown, respectively.

It have been reported that H3.3/H2A.Z double variant-containing nucleosomes are unstable *in vivo* (38), and they are positioned at the NDRs of active promoters and at the CBSs (39). To study whether H2A.Z plays a cause role in regulating the unwrapping states of nucleosomes, we performed MNase-X-ChIP-seq for H3.3-HA after H2A.Z knock down by siRNA in a mES cell line, where a HA tag is knocked-in at the C-terminal of *H3F3B* gene locus (20). In wild type cells, we found that H3.3 nucleosomes have higher ratio of small DNA fragments (30–80 bp) than large DNA fragments (140–168 bp) (Figure 4A), showing similar unwrapping states with H2A.Z nucleosomes. After H2A.Z depletion, the efficiency of siH2A.Z is confirmed by western blot (Supplementary Figure S4A), we found that the ratio of large DNA fragments of the input increased (Supplementary Figure S4B), and the unwrapping states of H3.3 nucleosomes changed dramatically, with increased ratio of large DNA fragments (120–168 bp) and decreased ratio of smaller DNA fragments (30–120 bp) (Figure 4A). These results suggest that H2A.Z maintains the unwrapping states of H3.3 nucleosomes, probably through the form of H2A.Z/H3.3 double variant-containing nucleosomes. Moreover, we observed a same trend of the changes of the unwrapping states of H3 nucleosomes as H3.3 nucleosomes, although much more mildly (Figure 4B and Supplementary Figure S4C). The reason for this difference may be that the ratio of H2A.Z/H3.3 nucleosomes over H3.3 nucleosomes is higher than the ratio of H2A.Z/H3 nucleosomes over H3 nucleosomes.

We further analyzed the unwrapping states of H3.3 nucleosomes at the TSSs and CBSs. We found that, unlike H2A.Z nucleosomes (Figure 2A and Supplementary Figure S2A), while large DNA fragments (140–168 bp) of H3.3 nucleosomes show enrichment at +1 nucleosomes and positive correlation with gene expression, the small DNA fragments (30–120 bp) of H3.3 nucleosomes are not (Figure 4C and Supplementary Figure S4D). After H2A.Z depletion, the signals of the large DNA fragments of H3.3 nucleosomes increased around TSSs, especially at +1 nucleosomes (Figure 4D and Supplementary Figure S4D). Compared with H3.3 nucleosomes at TSSs, H3.3 nucleosomes show different unwrapping states at the CBSs. Both small DNA fragments and large DNA fragments of H3.3 nucleosomes are well-phased around CBSs (Figure 4E), with highest signal of small DNA fragments (30–80 bp) (Supplementary Figure S4E). Similar to H2A.Z nucleosomes, unwrapped H3.3 nucleosomes with 30–80 bp DNA fragments also show a bimodal pattern around the CBSs (Figure 4E and Supplementary Figure S4E). After H2A.Z depletion, the large DNA fragments of H3.3 nucleosomes around CBSs increased (Figure 4F and Supplementary Figure S4E). These dynamic changes of unwrapping states of H3.3 nucleosomes at TSSs and CBSs suggest that H3.3 nucleosomes are less unwrapped after H2A.Z depletion, supporting that H2A.Z regulates the unwrapping states of H3.3 nucleosomes.

### H2A.Z regulates nucleosome unwrapping and CTCF binding

**Figure 5.**
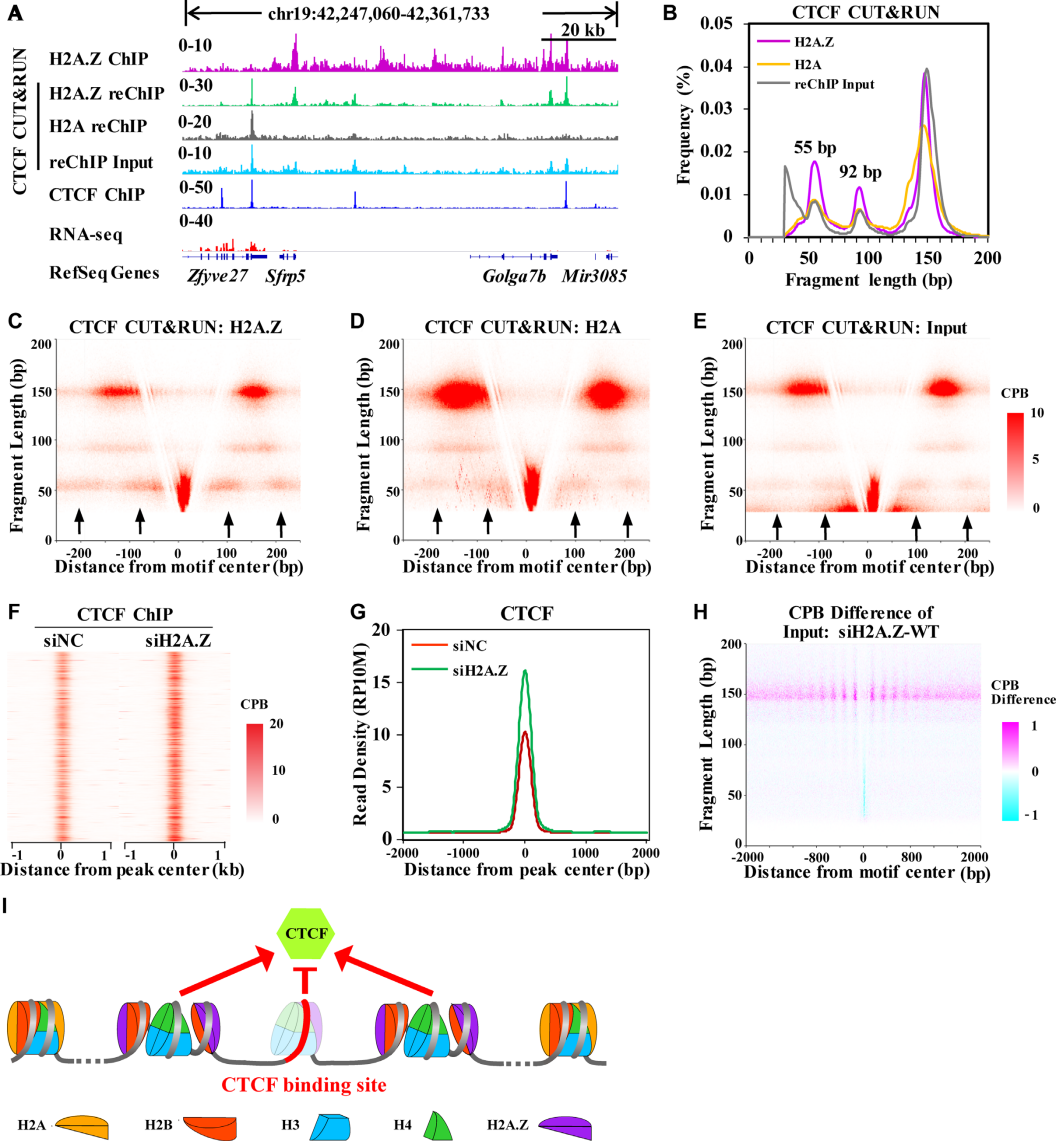
H2A.Z regulates nucleosome unwrapping and CTCF binding. (**A**) Genome tracks show the signals of H2A.Z ChIP-seq, H2A.Z or H2A re-ChIP after CTCF CUT&RUN, CTCF CUT&RUN and CTCF ChIP-seq. (**B**) Histograms show the FLPs of H2A.Z or H2A re-ChIPed DNA after CTCF CUT&RUN and total DNA of CTCF CUT&RUN. (C–E) V-plots show the distribution of fragments of H2A.Z (**C**) or H2A (**D**) re-ChIPed DNA after CTCF CUT&RUN and total DNA of CTCF CUT&RUN (**E**) around CBSs. (F, G) Heatmap (**F**) and meta-profile (**G**) and show the dynamics of CTCF around CBSs after H2A.Z knockdown. (**H**) A difference-V-plot shows the dynamics of DNA fragments of MNase-X-ChIP input at CBSs after H2A.Z knockdown. (**I**) A diagram shows the working model that unwrapped H2A.Z nucleosomes flanking CBSs facilitate CTCF binding while unwrapped H2A.Z nucleosomes at the center of CBSs compete with CTCF for binding. In unwrapped H2A.Z nucleosomes immediately flanking CBSs, the H2A.Z–H2B dimers may have detached from the (H3–H4)_2_ tetrasome to form open nucleosomes.

CUT&RUN has recently been developed to map chromatin binding factors (CBF) with higher signal to noise ratio compared with ChIP-seq (21). We re-analyzed the FLPs of nucleosomes released during CTCF CUT&RUN and H2A CUT&RUN reported by (21). We found that the digestion of nucleosomes released from CTCF CUT&RUN is limited over ∼300-fold time-course digestion range (Supplementary Figure S5A); however, the digestion of H2A nucleosomes during H2A CUT&RUN is time-dependent (Supplementary Figure S5B). Thus, the unwrapping states of nucleosomes released during CTCF CUT&RUN maybe more native than that of nucleosomes released during H2A CUT&RUN. To this end, we firstly performed CTCF CUT&RUN, then performed H2A.Z or H2A ChIP-seq using the released nucleosomes. We found that our CTCF CUT&RUN signal (‘re-ChIP input’) is similar with CTCF ChIP-seq (Figure 5A and Supplementary Figure S5C), confirming the specificity of CTCF CUT&RUN. We also found that H2A.Z re-ChIP signals and H2A re-ChIP signals showed different pattern (Figure 5A). Moreover, that the FLP of re-ChIP input showed enrichment of DNA fragments smaller than 50 bp, which is absent from those of H2A.Z re-ChIP and H2A re-ChIP (Figure 5B). These results suggested that the non-specific immunoprecipitation of CTCF bound DNA during re-ChIP, if any, is minimal. Importantly, the FLPs of both H2A.Z re-ChIP and H2A re-ChIP showed two sub-peaks at 55 bp and 92 bp (Figure 5B), apart from the ∼150 bp major fragment length peak, suggesting that both H2A.Z nucleosomes and H2A nucleosomes have unwrapping states in mES cells, besides the intact nucleosome structure. However, as these unwrapped H2A nucleosomes are released from chromatin regions where there is concurrent CTCF binding, it may be under represented during bulk MNase-X-ChIP-seq of H2A. V-plot analyses revealed that, for H2A.Z and H2A re-ChIP signals and CTCF CUT&RUN input, the DNA fragments around 55 bp showed bimodal distribution at each of the nucleosomes immediately flanking the CBSs (Figure 5C–E, indicated by black arrows). This result confirms the bimodal signals of unwrapping H2A.Z nucleosomes immediately flanking CBS, and the unwrapping of H2A.Z nucleosomes under native condition in mES cells.

To explore whether H2A.Z regulates the CTCF binding, we performed crosslink ChIP-seq for CTCF after depletion of H2A.Z. Interestingly, we found that the CTCF binding is globally increased after H2A.Z knockdown (Figure 5F, G), suggesting that the unwrapped H2A.Z nucleosomes around the CBSs may facilitate CTCF binding. This hypothesis supported by the increased signals of the large fragments (∼150 bp) at the flanking nucleosome positions of CBSs after H2A.Z knockdown, whereas the signals of smaller fragments did not change much (Figure 5H). However, the increased CTCF binding after H2A.Z knockdown may also arise from the loss of competition from the unwrapped H2A.Z nucleosomes at the center of CBSs. Taken together, as indicated in Figure 5I, our results showed that H2A.Z can regulate CTCF binding, probably through modulating the unwrapping states of nucleosomes at the CTCF binding regions.

## DISCUSSION

### Exploring the unwrapping of nucleosomes *in vivo* by MNase-X-ChIP-seq

In this study, through analysis of FLP by X-MNase-ChIP-seq, we revealed that nucleosomes containing histone variant H2A.Z is more unwrapped than canonical nucleosomes. We also revealed that the function of +1 H2A.Z nucleosomes in transcription is correlated with the unwrapping states of H2A.Z nucleosomes. We further proved that H2A.Z is required to maintain the unwrapping state of H3.3 nucleosomes, and that H2A.Z is involved in regulating the binding of CTCF, probably through modulating the unwrapping states of nucleosomes. Thus, we have presented convincing evidences that MNase-X-ChIP-seq can be used to study the unwrapping states of nucleosomes *in vivo* when controlled properly. Meanwhile, we are aware that the FLPs derived by X-MNase-ChIP-seq are dependent on the digestion condition. Thus, fragment length observed under a certain digestion condition cannot be taken as direct evidence for specific unwrapping state of nucleosome. In addition, although the positioning and pattern of small DNA fragments of H2A.Z nucleosomes at +1 nucleosomes (Figure 2A) and CBSs (Figure 3B) suggest that these signals are not merely crosslink artifacts, we cannot totally rule out that the small DNA fragments ChIPed by histones contain DNA fragments bound by crosslinked chromatin binding factors (i.e. transcription factors), as it has always been a puzzle for crosslink ChIP-seq. As supporting evidence for the unwrapping of nucleosomes *in vivo*, we observed small DNA fragments (∼55 bp) re-ChIPed by H2A.Z after CTCF CUT&RUN under native condition (Figure 5C). In the future, probably we can combine dynamic changes of FLPs from multiple digestion conditions and additional biochemical data of the stoichiometry of histones within a nucleosome to model specific nucleosome unwrapping state *in vivo*.

### Regulation of nucleosome unwrapping by H2A.Z

H2A.Z is evolutionary conserved variant histone for H2A. It plays important roles in the regulation of high order chromatin structure, gene transcription, DNA replication, DNA repair and genome integrity (40–42). It's also critical for early embryonic development, lineage commitment of stem cells, as well as somatic cell reprogramming to pluripotency (43,44). However, whether the nucleosomes containing H2A.Z would have distinct structural features compared with canonical nucleosomes, and whether H2A.Z would regulate these biological processes through modulating the nucleosome structure and dynamics *in vivo* remain largely unknown. In this study, we found that histone variant H2A.Z is preferentially associated with nucleosome unwrapping, which suggested that H2A.Z plays critical roles in the regulation of nucleosome stability and integrity. This conclusion is further supported by previous observations that in human CD4+ T-cells, H2A.Z nucleosomes protect only ∼120 bp of DNA from MNase digestion (45), and that in Drosophila S2 cells, both homotypic and heterotypic H2Av nucleosomes are associated with abundant DNA fragments shorter than 147 bp (46). Spontaneous equilibrium conformational transition of nucleosomes could result in exposure and subsequent digestion of the buried nucleosomal DNA by MNase (47). The rate and extent of unwrapping of the nucleosomal DNA are affected by DNA sequence as well as by variations of histones, especially of histones H2A and H3, as they contact the terminal DNA segments (2). Thus, H2A.Z could affect the nucleosome structure and stability by modulating the interaction between the nucleosomal DNA and histone octamers. In addition, the nucleosomes containing histone variants can be differentially remodeled by the ATP-dependent chromatin-remodeling factors and histone chaperones (2). Therefore, the enrichment of small fragments by unwrapped H2A.Z nucleosomes could be attributed at least from either unwrapping of the nucleosomal DNA or remodeling of nucleosome by remodelers or chaperones. In addition, we found that although the major fragments associated with canonical histones were large fragments (140–168 bp), considerable fragments smaller than 140 bp were observed, indicating unwrapping dynamics of canonical nucleosomes *in vivo*. Together with other studies (13,18,46), our results supported that the genome-wide unwrapping are conserved across different species.

According to the classic nucleosome assembly pathway, the H3-H4 tetramer is firstly deposited to DNA to form a tetrasome intermediate, which protects about 80 bp DNA; then H2A–H2B heterodimers are added to the tetrasome intermediate to form an intact nucleosome, which binds about 146 bp DNA (2). If the second step is a rate limiting step of nucleosome assembly *in vivo*, we should expect more small fragments (e.g. ∼80 bp) associated with H3–H4 tetramer than H2A-H2B dimers. However, we observed that histones H2A and H3 have similar FLPs, indicating that H2A–H2B dimers may be tethered to the tetrasome intermediate during the nucleosome remodeling *in vivo*. Consistent with this hypothesis, we and others found that histone chaperone FACT can facilitate the tethering of H2A-H2B dimers onto the tetrasome immediately and prevent the loss of H2A–H2B dimers from the nucleosome (7,18,48). In addition, the nucleosome is rapidly reassembled and maturated after DNA replication or transcription (49,50). Thus, it would be great of interest to study the regulatory function of FACT on the nucleosome unwrapping dynamics during DNA replication or transcription in vivo by MNase-X-ChIP-seq.

### Regulation of transcription by unwrapped +1 H2A.Z nucleosomes

It has been appreciated for a long time that the nucleosome is a barrier to transcribing Pol II *in vitro*, and this barrier can be overcome when the nucleosome is destabilized by either high salt or ionic detergent (8,51,52). Within the cells, Pol II can transcribe through the nucleosomal DNA efficiently, suggesting that other factors are involved in the overcoming of the nucleosome barrier. It has been shown that histone variant H2A.Z, which is enriched at the +1 nucleosome in eukaryotes (53), can lower the barrier to Pol II (54). In addition, H2A.Z was also involved in transcription repression through modulating the nucleosome stability, mobility and positioning (55–57). In this study, we found that the Cluster-D unwrapped +1 H2A.Z nucleosomes associated genes are more active than the genes associated with +1 H2A.Z nucleosome in other unwrapping states. The Cluster-D unwrapped +1 H2A.Z nucleosomes have relatively higher ratio of 120–140 bp fragments, most likely representing the hexasome intermediate in which the contaction between one H2A.Z-H2B or H2A-H2B dimer and the nucleosomal DNA is lost. In agreement with this, it has been reported that the +1 hexasome with distal H2A-H2B dimer loss facilitates Pol II elongation in drosophila (18). Moreover, we also found that the Cluster-A unwrapped +1 H2A.Z nucleosomes, with the highest ratio of fragments smaller than 80 bp, are preferentially associated with less actively transcribed genes, such as the genes involved in neuron differentiation and signaling transduction. We hypothesize that the Cluster-A unwrapped +1 H2A.Z nucleosomes might prime these genes for activation under inductive conditions. Consistent with this hypothesis, we have previously shown that H2A.Z is enriched at the promoters of signaling (all-*trans* retinoid acid) response genes and set a chromatin structure to potentiate these genes for activation (58). Thus, as shown in Figure 2F, our results suggested that the functions of the +1 H2A.Z nucleosomes in transcription regulation depend on its unwrapping states.

### Regulation of CTCF binding by H2A.Z nucleosome unwrapping

CTCF mediated 3D genome organization is critical for the establishment and maintenance of cell identity (59). However, how the CTCF binding is epigenetically regulated at genome-wide to fulfill this function remains largely uncharacterized. In this study, we observed positive correlation between the CTCF binding and H2A.Z occupancy flanking CBS, suggesting that H2A.Z nucleosomes play a role in CTCF binding. It has been hypothesized that depositions of histone variants H2A.Z and H3.3 could prime a chromatin context that is promising for CTCF binding (60). Here, we showed that H2A.Z is preferentially associated with nucleosome unwrapping. Therefore, H2A.Z could create more dynamic nucleosome organization around the CBSs to facilitate CTCF binding. Most recently, it was reported that CTCF is lost from its binding sites during mitotic phase, and the surrounding nucleosome arrays are rearranged to occupy the CTCF binding sites (61). However, H2A.Z was maintained at the CBSs, possibly functioning as bookmarks to enable inheritance of CTCF binding potential throughout the cell cycle (61). Thus, H2A.Z could function as a placeholder for CTCF binding during mitotic phase, which facilitates efficient re-binding of CTCF when the cell entering the next G1 phase. Interestingly, we observed increased CTCF binding after depletion of H2A.Z, suggesting that H2A.Z may have additional function in the regulation of CTCF binding. Consistently, we found that unwrapped H2A.Z nucleosomes with small DNA fragments (<80 bp) are positioned at the CTCF binding sites, indicating that unwrapped H2A.Z nucleosomes and CTCF could dynamically compete with each other for binding to the CBSs (Figure 5I). It is reported that CTCF dynamically scans the genome for cognate binding sites and transiently bind chromatin with residence time between 1 and 2 min (62,63). Moreover, CTCF binding can be prevented by repositioning of nucleosomes over the CTCF binding sites during transcriptional activation (64). Thus, unwrapped H2A.Z nucleosomes positioned at the CTCF binding sites could function as rheostat for CTCF binding outside mitotic phase. Taken together, our results and others suggest a dual function model for unwrapped H2A.Z nucleosomes in the regulation of CTCF binding. During mitotic phase, unwrapped H2A.Z nucleosomes around the insulators function as a placeholder for CTCF and create a promising chromatin context favoring CTCF re-binding. Outside mitotic phase, the unwrapped H2A.Z nucleosomes at the CBSs function as rheostat to repress overloading of CTCF.

## DATA AVAILABILITY

The datasets generated and analyzed during the current study are submitted to the Gene Expression Omnibus (GEO) repository under the GEO accession GSE146082. Mouse ES cell gene expression data and total Pol II ChIP-seq data were downloaded from GSE117035 (20). H3K4me3 and H3K27ac data were downloaded from GSE48519 and re-analyzed to annotate the cluster of +1 H2A.Z nucleosomes. The genomic coordinates of peaks of H3K4me1, H3K4me3, H3K27ac, H3K36me3 and H3K27me3 in mouse ES cells ES-Bruce4 were downloaded from the ENCODE project (https://www.encodeproject.org/). CTCF and H2A CUT&RUN data were downloaded from GSE84474. Coordinates for transcription state sites (TSSs) were retrieved from RefSeq Genes (mm9) downloaded from UCSC genome browser (http://genome.ucsc.edu/cgi-bin/hgTables). The code used for V-plot analyses is available at https://github.com/WenZengqi/nucleosome_unwrapping. The materials used during the current study are available from the corresponding author on reasonable request.

## Supplementary Material

gkaa360_Supplemental_Files
