# Supplementary material for: Histone variant H2A.Z regulates nucleosome unwrapping and CTCF binding in mouse ES cells
Source: Nucleic Acids Res. 2020 May 11;48(11):5939–52. doi: 10.1093/nar/gkaa360 (PMC7293034; doi:10.1093/nar/gkaa360)
Supplement: gkaa360_Supplemental_Files [file gkaa360_supplemental_files.zip › H2A.Z regulates nucleosome unwrapping-Supplementary Material.pdf]

Histone variant H2A.Z regulates nucleosome unwrapping and CTCF binding in mouse ES cells

Zengqi Wen[1,2*], Liwei Zhang[1], Haihe Ruan[1*] and Guohong Li[1,2*]

[1]National Laboratory of Biomacromolecules, CAS Center for Excellence in Biomacromolecules, Institute of Biophysics, Chinese Academy of Sciences, Beijing 100101, China; [2]University of Chinese Academy of Sciences, Beijing, China

[*]Correspondence: liguohong@sun5.ibp.ac.cn; ruanhh@ibp.ac.cn; wen_zengqi@moon.ibp.ac.cn

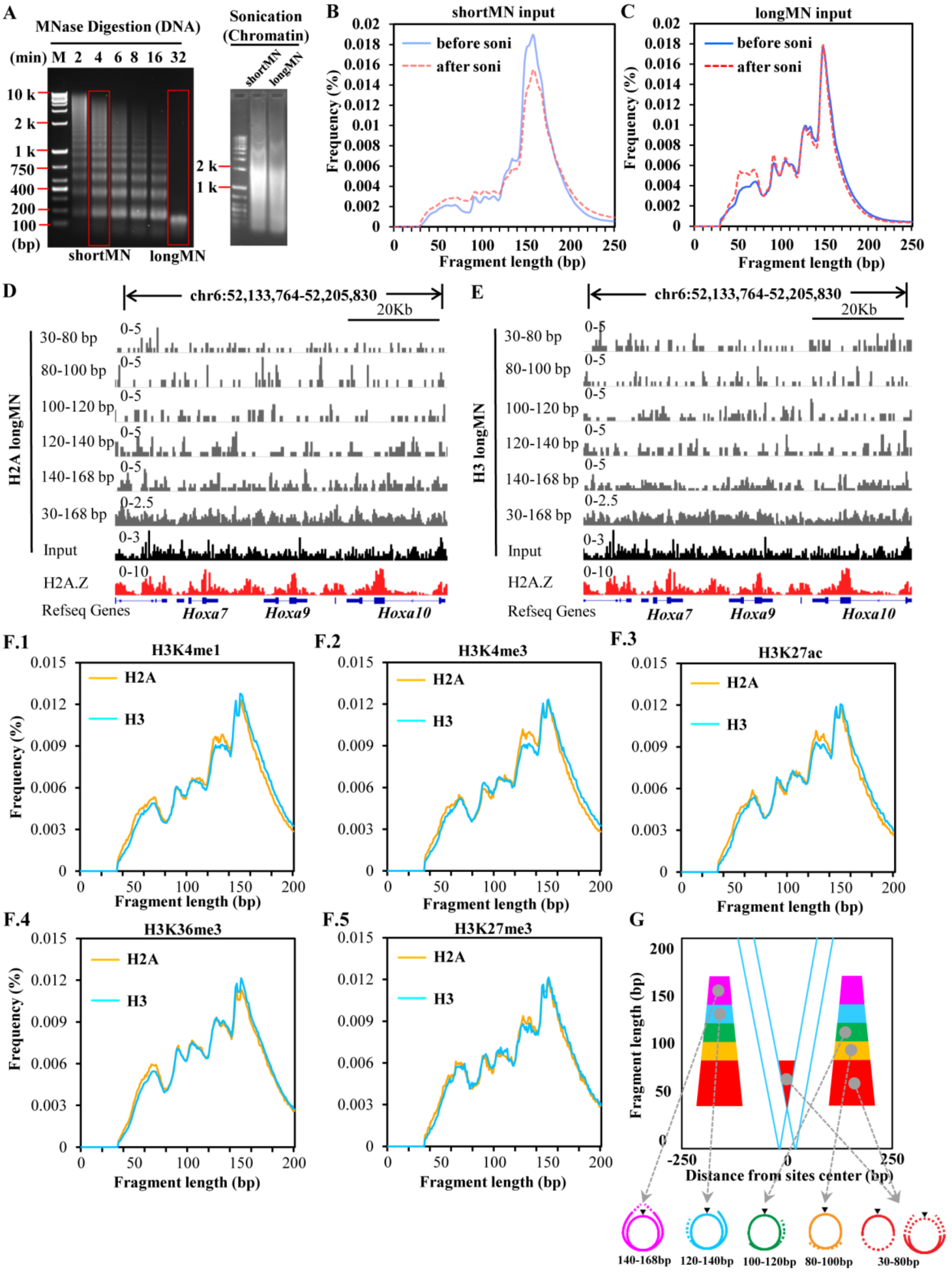

Figure S1. H2A.Z is enriched with nucleosome unwrapping.

(A) Left panel: agarose gel shows the DNA length after time-course MNase digestion. The 4 min digestion and 32 min digestion conditions were referred as “shortMN” and “longMN”, respectively. Right panel: sonicated chromatin from “shortMN” and “longMN” were directly analyzed by agarose gel. The size range of chromatin fragments is same as for regular ChIP.

(B, C) Histograms show the FLPs of chromatin from “shortMN” (B) and “longMN” (C) before and after sonication.

(D, E) Genome tracks show the distribution of H2A (D) or H3 (E) ChIPed fragments under

"longMN" digestion conditions. The signals for all the tracks are normalized by reads per million (RPM) per 10bp bin.

(F) Histograms show the FLPs of DNA fragments ChIPed by H2A or H3 histones at 1 kb regions around the peaks of H3K4me1, H3K4me3, H3K27ac, H3K36me3 and H3K27me3.

(G) A diagram shows the interpretation of DNA fragments in the V-plot. The X-axis indicates distance from the middle point of a DNA fragment to the binding sites center, i.e. TSSs or CBSs. The direction of binding sites are arranged that the 5' upstream of binding sites on either plus strand or minus strand is at left. The Y-axis indicates the length of DNA fragments protected by the nucleosomes positioning besides the binding sites. Thus, the value of each dot in this two-dimensional space represents the number of DNA fragments with corresponding length at Y-axis and located at corresponding distance at X-axis from the binding site center. The value for the matrix is normalized as centers of DNA fragments per billion reads (CPB). The magenta, cyan, green, orange and red trapezoids represent nucleosomes with 140-168bp, 120-140bp, 100-120bp, 80-100bp and 30-80bp DNA, respectively, positioned nearby the binding sites. The red triangle represents unwrapped nucleosomes with 30-80bp DNA located at the binding sites. The protected DNA and digestion path of MNase for each unwrapping states of nucleosome is illustrated by the solid lines and dash lines of the colored circles below, respectively.

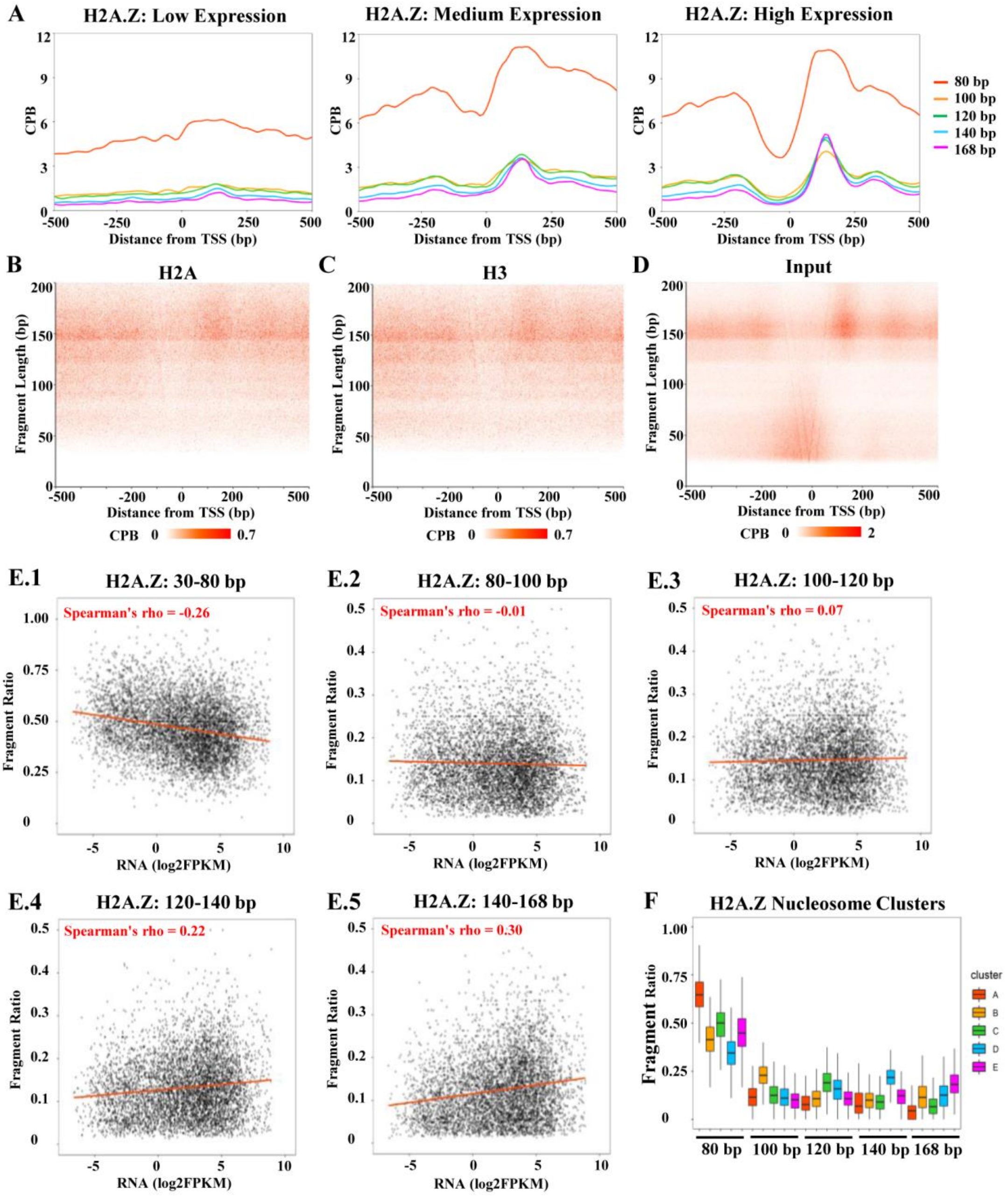

Figure S2. Unwrapping of H2A.Z nucleosomes at the promoters.

(A) Meta profiles show the reads density of the five fragment groups of H2A.Z ChIPed DNA around 1 kb regions of TSSs of low, medium and high expressed genes.

(B-D) V-plots show the fragment length distribution of H2A (B) or H3 (C) ChIPed DNA and input of MNase-X-ChIP-seq (D) around 1 kb regions of TSS.

(E) Scatter plots show the correlation between gene expression and the ratio of 30-80 bp (E.1), 80-100 bp (E.2), 100-120 bp (E.3), 120-140 bp (E.4) and 140-168 bp (E.5) fragments within +1 H2A.Z nucleosomes. Red lines represent the general linear regression.

(F) Boxplots show the ratio of the five DNA fragment groups of cluster A-E +1 H2A.Z nucleosomes.

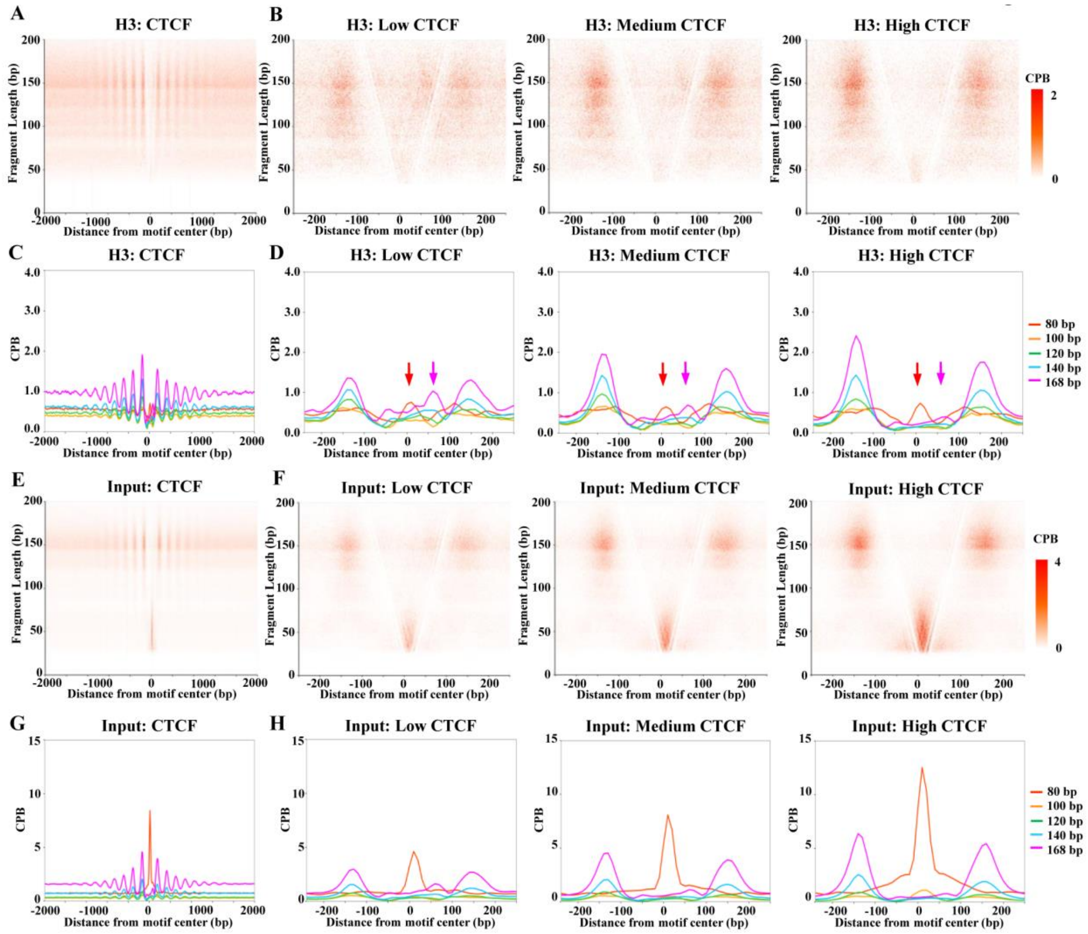

Figure S3. Unwrapping of H2A.Z nucleosomes at the CTCF binding sites.

(A, E) V-plots show the FLPs of H3 (A) ChIPed DNA or input of MNase-X-ChIP-seq DNA (E) around 4 kb regions of CBS sites.

(B, F) V-plots show the FLPs of H3 (B) ChIPed DNA and input of MNase-X-ChIP-seq DNA (F) around 500 bp regions of CBS sites with low, medium and high CTCF binding.

(C, G) Meta profiles show the reads density of the five fragment groups of H3 (C) ChIPed DNA and input of MNase-X-ChIP-seq DNA (G) around 4 kb regions of CBS sites.

(D, H) Meta profiles show the reads density of H3 (D) ChIPed DNA and input of MNase-X-ChIP-seq DNA (H) around 500 bp regions of CBS sites with low, medium and high CTCF binding. Red arrows in Figure D indicate moderate signals of unwrapped H3 nucleosomes with 30-80bp DNA at the center of CBSs. Cyan arrows in Figure D indicate moderate signals of intact H3 nucleosomes between CBSs the downstream nucleosome.

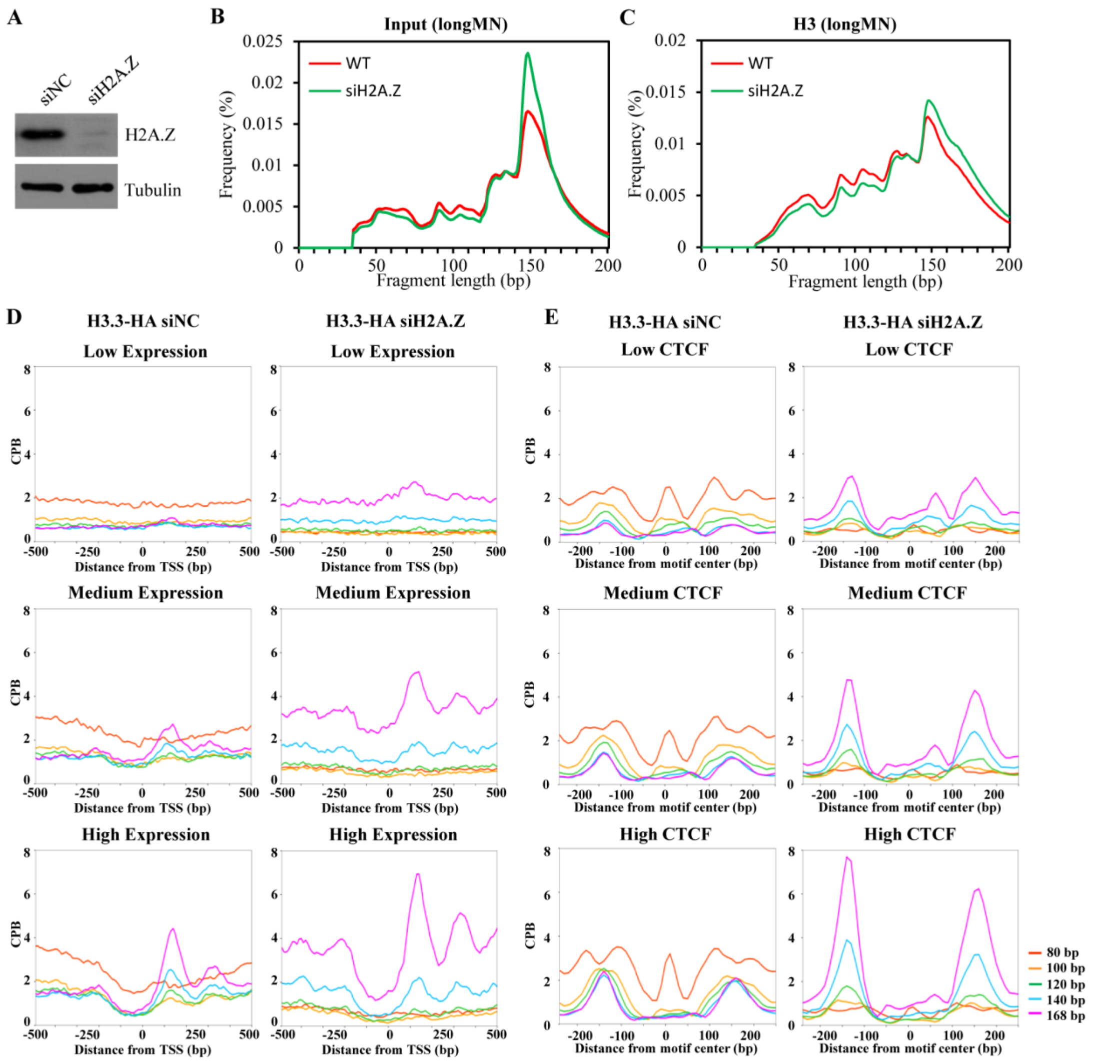

Figure S4. H2A.Z regulates the unwrapping of H3.3 nucleosomes.

(A) Western blot show the efficiency of H2A.Z knockdown. Tubulin is used as loading control.

(B-C) Meta-profiles show the FLPs of input DNA (B) or H3 ChIPed total DNA (C) in both wild type and H2A.Z knockdown cells.

(D-E) Meta profiles show the changes of reads density of the five fragment groups of H3.3-HA ChIPed DNA after H2A.Z knockdown, around TSSs of low, medium and high expressed genes (D), or around CBSs with low, medium and high CTCF binding (E).

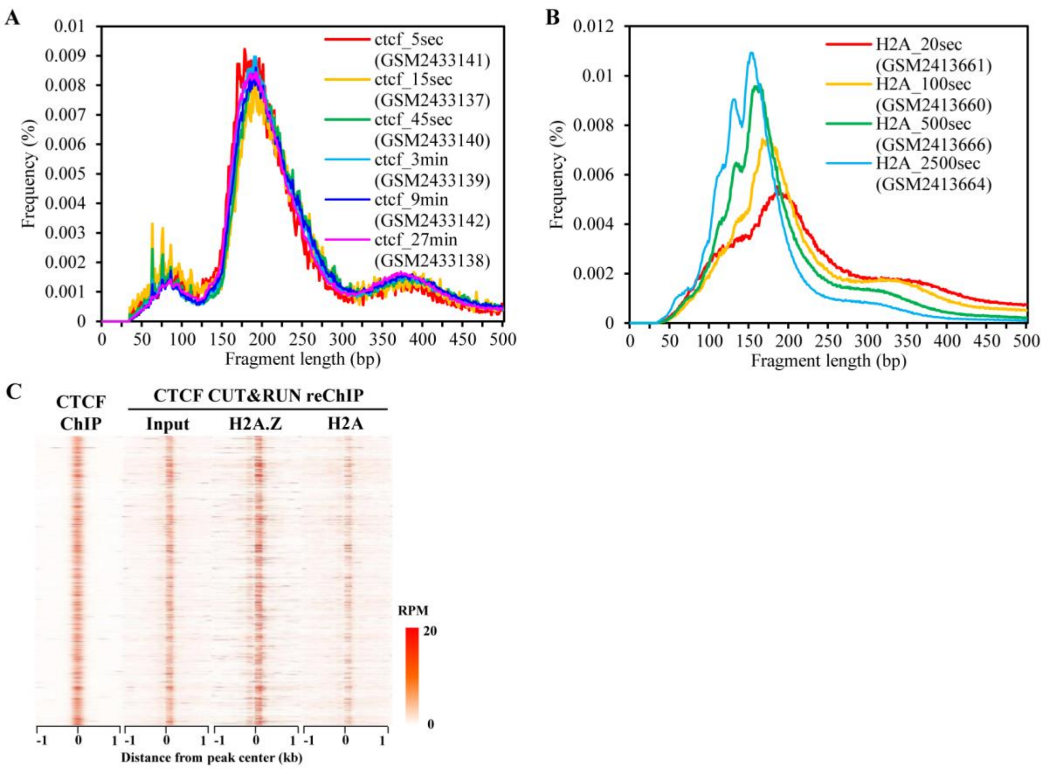

Figure S5. H2A.Z regulates nucleosome unwrapping and CTCF binding.

(A, B) Meta-profiles show the FLPs of nucleosomes released during time-course CTCF CUT&RUN (A) or FLPs of nucleosomes during H2A time-course CUT&RUN. The CUT&RUN data were reported in (Skene and Henikoff, 2017), and were downloaded from the Gene Expression Omnibus (GEO) repository under the GEO accession GSE84474.

(C) Heatmap shows the signals of CTCF ChIP, CTCF CUT&RUN, H2A.Z and H2A re-ChIP after CTCF CUT&RUN.

Supplemental table 1. Cluster A H2A.Z nucleosome molecular function: The genes associated with cluster A H2A.Z nucleosomes were subjected to Gene Ontology analysis using HOMER. GO terms with 10-500 target genes and p-value < 0.01 were displayed in Figure 2E.

Supplemental table 2. Cluster D H2A.Z nucleosome molecular function: The genes associated with cluster D H2A.Z nucleosomes were subjected to Gene Ontology analysis using HOMER. GO terms with 10-500 target genes and p-value < 0.01 were displayed in Figure 2E.

Supplemental table 3. Cluster A H2A.Z nucleosome motif: The promoter regions (from 300 bp upstream of TSSs to 50 bp downstream of TSSs) of genes associated with cluster A H2A.Z nucleosomes were subjected to motif analysis using HOMER. Motifs with p-value < 0.01 were selected.

Supplemental table 4. Cluster D H2A.Z nucleosome motif: The promoter regions (from 300 bp upstream of TSSs to 50 bp downstream of TSSs) of genes associated with cluster D H2A.Z nucleosomes were subjected to motif analysis using HOMER. Motifs with p-value < 0.01 were selected.
